# Rhubarb-Evoke Mucus Secretion through Aggregation and Degranulation of Mast Cell in the Colon of Rat: In vivo and ex vivo studies

**DOI:** 10.1038/s41598-019-55937-7

**Published:** 2019-12-18

**Authors:** Di Wu, Xiaowei Xue, Chenchen Gao, Yuehong Liu, Tiantian Wang, Lisheng Li, Xuehong Tong, Feng Li, Jingdong Xu

**Affiliations:** 1 Department of Physiology and Pathophysiology, School of Basic Medical Science, Capital Medical University, Beijing, 100069 China; 2 Key laboratory of Carcinogenesis and Translational Research (Ministry of Education/Beijing), Department of Interventional Therapy, Peking University Cancer Hospital & Institute, Beijing, 100142 China; 3 Department of Pathology, Peking Union Medical College Hospital, Chinese Academy of Medical Sciences & Peking Union Medical College Beijing, Beijing, 100730 China; 4 Department of Radiology, Xuanwu Hospital, Capital Medical University, Beijing, 100053 China; 5 Experimental Center for Basic Medical Teaching, School of Basic Medical Science, Capital Medical University, Beijing, 100069 China; 6 Department of Neurobiology, School of Basic Medical Science, Capital Medical University, Beijing, 100069 China

**Keywords:** Physiology, Gastroenterology

## Abstract

Rhubarb is commonly used to treat constipation in China for its function of promoting intestinal movement and optimum water content in feces. However, its mechanism of mucus secretion is vague. The aim of the study is to investigate the role of mast cells and enteric neurons in rhubarb extract (RE)-induced mucus secretion in the rat colon. Immunofluorescence was used to detect histamine receptors. Western blotting and 3,3′-diaminobenzidine (DAB) were applied to explore the content changes of mast cells activation. The changes in colonic goblet cells (GCs) were determined by means of PAS/AB staining. An intestinal perfusion system with a Bradford protein assay kit was directly to estimate *in vitro* secretion. And the cytokines were investigated with ELISA. The longitudinal aspect of this study indicate that the number and water content of faecal pellets were enhanced after the administration of different doses of RE accompanied by mast cells accumulated and increased the content of interferon (IFN) -γ or decreased the levels of interleukin (IL) −10 at doses of 3 and 6 g/kg. Pretreatment with ketotifen, mast cell stabilizer, had partially inhibited on RE-induced mucus secretion. Furthermore, RE induced the release of acetylcholine and mucin-2 in the colonic tissue and the histamine levels from the faeces. The results suggest that RE induced colonic mucus secretion involves mast cell activation and some cytokine.

## Introduction

Rhubarb, as a species of *Polygonaceae*, has been clinically used for fever, diarrhoea, detoxification and detumescence in Traditional Chinese medicine (TCM) for thousands of years. Besides being used as a laxative, rhubarb also plays an important role in protecting the gut barrier, maintaining the intestinal micro-ecological environment and preventing bacterial translocation^1,2^. The main roles of rhubarb are attributed to its 2–5% content of anthraquinone derivatives^3^, which include rhein, emodin, chrysophanol, aloe-emodin, and physcion^4^. Rhubarb has been found to increase intestinal water content and ion secretion, which may contribute to relieve constipation^5^. Furthermore, anthraquinone as the main active ingredient of rhubarb root extract, augments rat ileal contraction by triggering release of endogenous acetylcholine^6^. Our previous study showed that emodin stimulates rat colonic epithelial Cl^−^ secretion, which is predominantly mediated by endogenous prostaglandin release and histamine^7^. At present, preparations containing rhubarb in chinese pharmacopoeia only achieve purgative efficacy for its free anthraquinones with its stimulate the colon plexus and inhibit Na^+^-K^+^-ATPase^8^. As it is well known that most mucin secreted by goblet cells (GCs) produced in the gastrointestinal tract, a diverse family of densely glycosylated proteins with its key characteristic ability to form gels, protect intestinal tract against infectious agents^9,10^ and facilitate movement.

As mucins play a key role in the protection of the underlying epithelium, any quantitative change in mucus secretion may modify this defensive barrier and have important physiological implications. As GCs migrate up the colonic crypt, they synthesize, store, and secrete mucin-2 via regulate secretion^11^. Endogenous factors, such as acetylcholine (ACh) and serotonin, can trigger mucin-2 secretion from colonic GCs^12,13^. However, the effect of RE on colonic mucus secretion remains blurry. In this study, we aimed to investigate the RE conduct mucus secretion of colonic GCs with its underlying mechanism.

## Results

### Effects of short-term RE administration on faeces in rats

**Figure 1:**
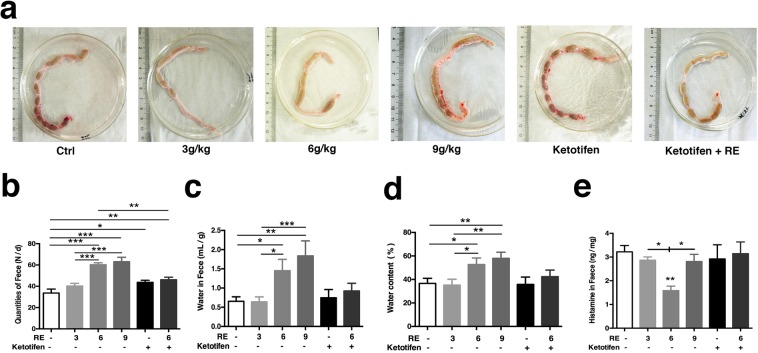
Effect of ketotifen on RE-evoked colonic faeces changes. (**a**) Concentration-response relationship for RE -evoked ahead or treatment with ketotifen for 24 h on faeces. (**b**,**c**,**e**) The bar graph illustrates the quantities of faeces (F = 16.02, P < 0.0001) and the water (F = 4.063, P = 0.003) and histamine content (F = 2.638, P = 0.347) in feces after administration of RE alone and treatment with ketotifen. (**d**) Moisture accounts for the wet weight of feces in the different groups (F = 3.319, P = 0.01). Data are presented as the mean ± S.E.M. *P < 0.05, **P < 0.01, ***P < 0.001.

To investigate the effect of RE to increase the water excretion after the administration of RE to rats, the faecal granule number and faecal water content were determined over 24 h. Compared with that of the control group, the faecal water content in the group treated with 3 g/kg RE increased from 0.66 ± 0.12 mL/g (n = 11) to 0.64 ± 0.13 mL/g (p > 0.05, n = 11); in the groups treated with 6 g/kg and 9 g/kg RE, the respective faecal water content values increased to 1.45 ± 0.30 mL/g (p = 0.025, n = 11), and 1.84 ± 0.40 mL/g, (p = 0.001, n = 11). After pretreatment with ketotifen, the water content exhibited no obvious change, with a value of 0.75 ± 0.21 mL/g (p > 0.05, n = 11). After pretreatment with ketotifen 1 h later and treatment with RE at a dose of 6 g/kg subsequently, the water content was increased compared to that of the control group, with a value of 0.92 ± 0.20 mL/g (p > 0.05, n = 11). The faecal pellet number exhibited the same trend as the water content, as shown in Fig. 1a–c. The number of particles excreted from faeces exhibited the same trend as the water content, as shown in Fig. 1a–c. Ratio of moisture content for the weight of faeces in the control were 36.66%. With 3 g/kg, 6 g/kg and 9 g/kg RE given, the ratio of water content in faeces showed an increasing trend as 35.17%, 52.72%, 58.00% respectively, which has no difference ratio of water content in faeces between the control group and the group with treatment ketotifen. Administration ketotifen 1 h and addition with RE at a dose of 6 g/kg subsequently, the ratio of the water content in faeces is up to 42.35% (Fig. 1d).

At same time, the content of histamine in the faeces decreased from 3.22 ± 0.27 ng/mg (n = 9)in the control group to 2.87 ± 0.14 ng/mg, which represents a decrease of 10.86% (p > 0.05, n = 9), in the group treated with 3 g/kg RE. In addition, this value decreased to 1.58 ± 0.19 ng/mg, which represents a decrease of 50.93% (p = 0.003, n = 9), in the group treated with 6 g/kg RE. However, no significant decrease, with a value of 2.81 ± 0.30 ng/mg and a reduction of 12.73% (p > 0.05, n = 9), was observed in the group treated with 9 g/kg RE. After pretreatment with ketotifen, the content of histamine in the faeces decreased to 2.92 ± 0.59 ng/mg, which represents a reduction of 9.31% (p > 0.05, n = 9). However, this value was 3.14 ± 0.49 ng/mg, with a slightly increasing trend (p > 0.05, n = 9), in the group treated with ketotifen and RE (Fig. 1e). From all these results, we could speculate that RE may increase defecation, and histamine released from mast cells might involve in this process.

### Distribution and quantification of mast cells in the colon after the administration of RE in rats

**Figure 2:**
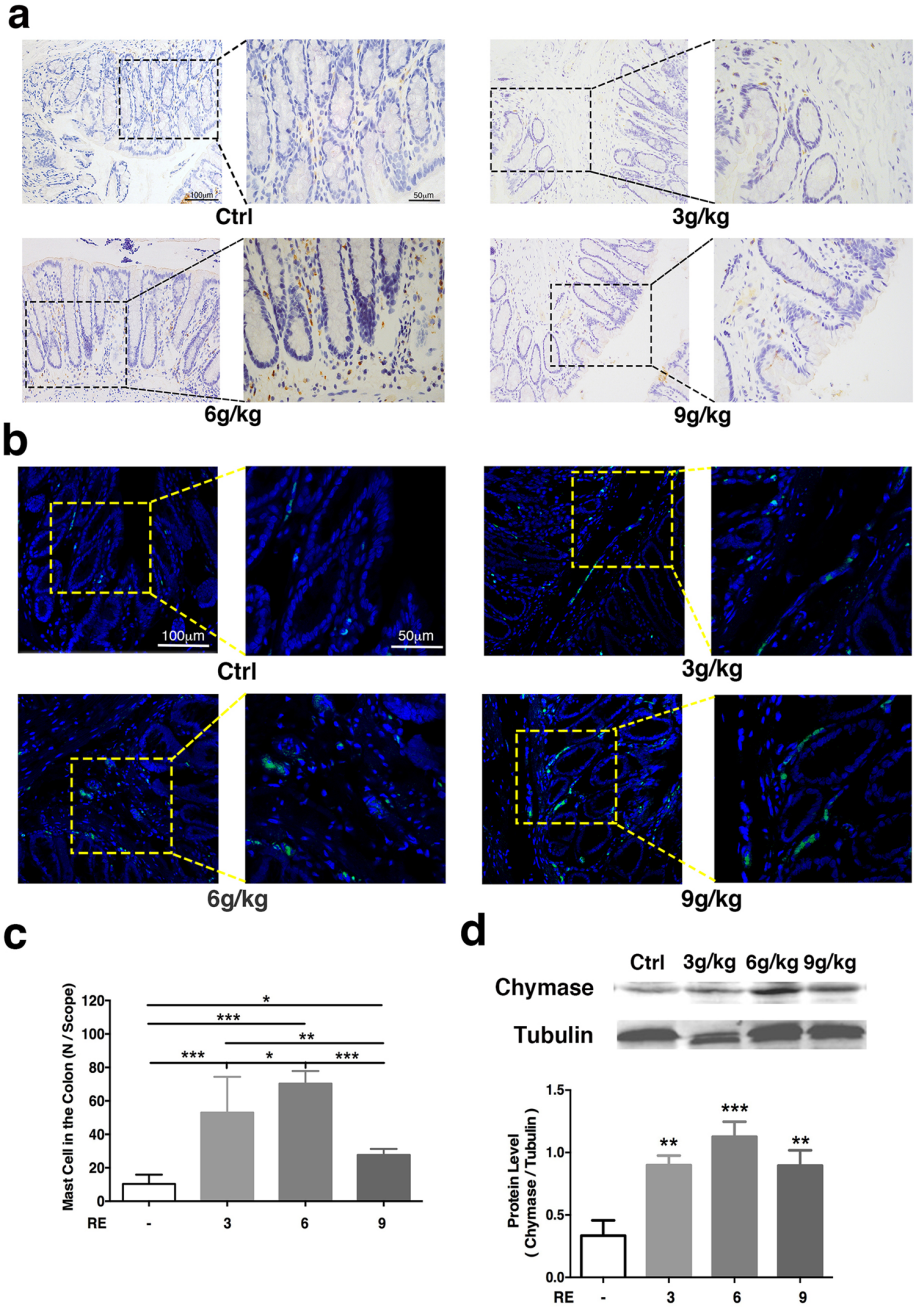
Immunohistochemistry detection of the mast cells on the rat distal colon. (**a**) Changes in the number of CD117^+^ mast cells with DAB staining in the rat colon. (**b**) Changes in mast cells observed with immunofluorescence in the rat colon. Mast cell chymase is shown in green. (**c**) The bar graph illustrates the changes in mast cells number with different doses of RE alone by means of DAB. (F = 30.45, P < 0.0001). (**d**) Western blotting quantified the expression of mast cell chymase in the rat colon. Densitometric analysis of chymase protein levels normalized to β-tubulin. Data are presented as the mean ± S.E.M. *P < 0.05, **P < 0.01, ***P < 0.001.

Just as previous studies have reported that rhubarb can cause degranulation of mast cells^14^, which could enhance transepithelial water secretion by acting on H receptor^15^. In order to exam the changes of the distribution and quantification of mast cells in the colon after the administration of RE in rats, as shown in Fig. 2a,c, the migration and accumulation of mast cells were observed in the colon tissue specimen after DAB staining and the detection of CD117 positive cell, mast/stem cell growth factor receptor (SCFR), which is a receptor tyrosine kinase that is also known as proto-oncogene c-Kit or tyrosine protein kinase Kit^16^. The number of CD117 positive cells increased from 10.33 ± 2.26 (n = 8, 200×) in the control group to 53.00 ± 8.73 in the group treated with 3 g/kg RE, which represents an increase of 413.06% (p < 0.001, n = 8, 200×), and 70.33 ± 3.07 (p < 0.001, n = 8, 200×) in the group treated with 6 g/kg RE, about 580.83% increase. Although the increase in the group treated with 9 g/kg RE was not as large as the increases observed in the other two groups, the value was to 27.67 ± 1.48 (p = 0.019, n = 8, 200×), also enhancement about 167.86%. The results obtained from immunofluorescence and Western blotting were consistent with the results of DAB staining (Fig. 2b,d). As expected, the levels of chymase, a marker of activated mast cells, was significantly enhanced about 80.05% (p = 0.003, n = 11), 137.11% (p < 0.001, n = 10) and 91.64% (p = 0.003, n = 9), at doses of 3 g/kg, 6 g/kg and 9 g/kg RE, respectively. These results showed that RE might play a potential role in promoting the aggregation and activation of colonic mast cells in rats.

### Change in GC quantity and colonic mucus secretion after administration of RE or ketotifen *in vivo*

We observed the changes in mucus secretion and GC activity that occurred when different doses of RE were administered to rats. “Sentinel” goblet cells (senGCs) localize to the colonic crypt entrance. These cells endocytose nonspecifically and protect the colonic crypt from bacterial intruders that have penetrated the inner mucus layer to maintain colonic mucoid bilayer stability^17^.

**Figure 3:**
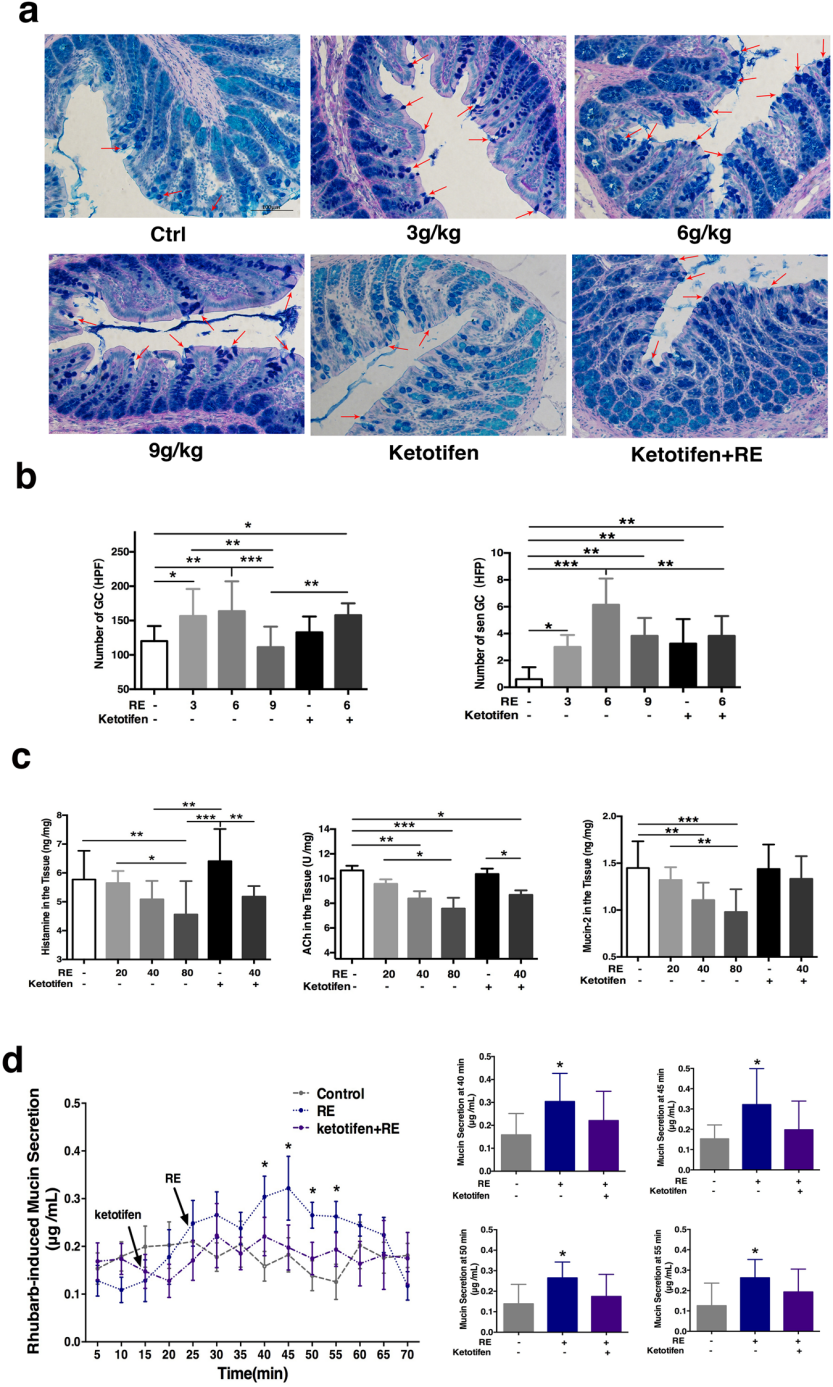
Pharmacology of RE-induced the change in GC number and content of mucus secretion. (**a**) Changes in GC number and mucin secretion by PAS/AB in the rat colon under a 200× field. Red arrows refer to senGCs. (**b**) The bar graph illustrates the change in GC (F = 4.978, P = 0.0008) and senGC (F = 8.219, P < 0.0001) numbers with different doses of RE ahead and treatment with ketotifen by PAS/AB under HPF. (**c**) RE-induced Ach (F = 5.032, P = 0009), histamine (F = 4.913, P = 0.001), Mucin-2 (F = 5.436, P = 0006) released from mucosa/submucosa preparations of the rat colon with different doses of RE and treatment with ketotifen. (**d**) Representative traces of RE effects on colonic secretion *in vitro* with the intestinal perfusion system and quantitative analysis using the Bradford protein assay kit. Data are presented as the mean ± S.E.M. *P < 0.05, **P < 0.01, ***P < 0.001.

In order to directly identify RE-involved in mucous secretion of goblet cells in colon, light microscopy was used to observe the intensity of staining and count the GCs under 200×magnification. As Fig. 3a shown, the results indicate that the number of GCs was 120.2 ± 6.34 high power field (HPF) (n = 12) in the control group. This value was significant enhanced to 156.6 ± 13.12 HPF by 30.0% (p = 0.012, n = 9), and to 163.7 ± 12.56 HPF about 35.83% (p = 0.002, n = 12), in the groups subjected to gavage administration of RE at doses of 3 g/kg and 6 g/kg, respectively. While the number of GCs slightly decreased with no significant change, to 111.3 ± 9.01 HPF of 7.40% (n = 11, p > 0.05), in the group subjected to gavage administration of RE at a dose of 9 g/kg. All these results suggest that RE may increase GC numbers. While the number of GCs was 132.8 ± 8.18 HPF (n = 8, p > 0.05) in the group of treatment ketotifen at the 0.30 g/kg, which was not obviously different from that of the control group, while administration with ketotifen 1 h ahead by gavage RE at a dose of 6 g/kg, the number of GCs is obviously increased to 157.8 ± 7.02 HPF (p = 0.021, n = 8) compared with the number observed after treatment with RE alone, The result imply that mast cells might be partly involved in this process. The number of senGCs was 0.60 ± 0.40 HPF (n = 12) in the control group. At the same time the SenGCs were also observed the SenGCs number was obviously enhanced from0.60 ± 0.40 HPF (n = 12) to 3.0 ± 0.37 HPF (p = 0.013, n = 8), 6.14 ± 0.74 HPF (p < 0.001, n = 8), and 3.83 ± 0.54 HPF (p = 0.001, n = 8) in the groups subjected to gavage administration of RE at doses of 3 g/kg, 6 g/kg and 9 g/kg, respectively. At same time, the number of senGCs enhanced to 3.25 ± 0.65 HPF (p = 0.004, n = 8) in the group subjected to gavage administration of ketotifen alone compared with that of the control group, which was also significant increase to 3.83 ± 0.60 HPF (p = 0.001, n = 8) in the group subjected to gavage administration of ketotifen ahead and RE at a dose of 6 g/kg subsequently(Fig. 3b). SenGCs exhibited changes similar to those observed for GCs and RE plays a role in GC mucus secretion.

### RE-induced ACh and histamine release *ex vivo*

**Figure 4:**
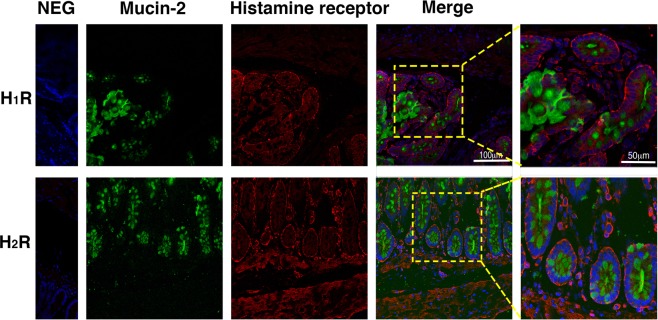
Location of Mucin-2 and HR with immunofluorescence in the rat colon Green fluorescence is mucin-2, and red fluorescence represents H_1_R and H_2_R, respectively. The eight pictures on the left are under a 400× field. The two pictures on the right are at 800× magnification. Scale as 100 µm and 50 µm.

As Fig. 4 shown, by immunofluorescence, it was noticed that there are much mucin-2 vesicles in the GC cytoplasm, and the H_1_ and H_2_ receptors are distributed in the colonic GC membrane, especially on the basal side. In order to determine whether RE could stimulate ACh and histamine release, the contents of ACh, histamine and mucin-2 in the colon tissue were measured with ELISA from different groups after incubation. As shown in Fig. 3c, under basal condition, the ACh content in the colonic tissues was 10.66 ± 0.38 U/mg (n = 9). When the colonic tissue was incubated at RE doses of 20 μg/ml, 40 μg/ml and 80 μg/ml, the ACh contents of the tissue decreased to 9.57 ± 0.37 U/mg about 10.20% (p > 0.05, n = 9); 8.38 ± 0.60 U/mg, by 21.39% (p = 0.004, n = 9); and 7.58 ± 0.87 U/mg by 28.93% (p < 0.001, n = 9), respectively. After pretreatment with ketotifen at the concentration of 100 μM, the ACh content was 10.37 ± 0.44 U/mg (p > 0.05, n = 9), with no significant change to the control group; in contrast, after pretreatment with ketotifen ahead and RE at a concentration of 40 μg/ml subsequently, the ACh content of the colon tissue decreased to 8.68 ± 0.37 U/mg by 18.60% (p = 0.012, n = 9) compared with the content of the control group.

As shown in Fig. 3c, pretreatment with RE at concentrations of 20 μg/mL, 40 μg/mL and 80 μg/mL, the histamine content is decreased from 5.77 ± 0.33 ng/mg (n = 9) to 5.65 ± 0.14 ng/mg about 2.07% (p > 0.05, n = 9); 5.09 ± 0.21 ng/mg about 11.79% (p > 0.05, n = 9); and 4.56 ± 0.44 ng/mg about 20.97% (p = 0.005, n = 9), respectively. The results indicated that the trend of histamine change was similar to that of Ach. In the colon preparations, ketotifen (100 μM) caused stronger reduction of RE (40 μg/mL)-induced histamine release from 6.40 ± 0.37 ng/mg (p > 0.05, n = 9), to and 5.18 ± 0.12 ng/mg (p > 0.05, n = 9) in pretreatment with ketotifen and RE at a concentration of 40 μg/m. These results suggest that RE -induced enhancement of histamine was partly mediated by mast cell activation.

It is well known that intestinal mucus, being high-molecular-mass glycoproteins, are secreted by GCs and form a highly hydrated mucus gel that coats the epithelial surface of the intestinal tract^18^. In order to further verify the efficacy of rhubarb in moistening intestines and relieving constipation, the content of mucin-2, the main component of mucus in the colonic tissues, was also measured. Under basal conditions, the mucin-2 content was 1.45 ± 0.10 ng/mg (n = 9). Similarly pretreatment with RE at concentrations of 20 μg/ml, 40 μg/ml and 80 μg/ml, resulted reduction in 8.77% (p > 0.05, n = 9) to 1.32 ± 0.05 ng/mg; 23.54% (p = 0.005, n = 8) to 1.11 ± 0.07 ng/mg, 38%(p < 0.001, n = 8) to 0.98 ± 0.09 ng/mg respectively. While pretreatment with ketotifen alone or ketotifen 10 mins later then adding RE at a dose of 40 μg/ml, the mucin-2 content in tissues was 1.44 ± 0.09 ng/mg (p > 0.05, n = 8) or 1.33 ± 0.09 μg/mg by19.82%(p > 0.05, n = 8), respectively.

To further test the hypothesis that GC- derived mucus is involved in RE-induced colonic mucus secretion, Elisa was used to measure mucus secretion from the rat colon sample following RE treatment. As shown in Fig. 3d, after an equilibration time of 40 min as the basal condition following RE treatment, mucin-2 accumulation in perfusate of the colon tissues was as control. Pretreatment of the tissues with RE at a concentration of 40 μg/ml in the perfusion chambers. There was a sustained enhancement of mucus secretion especially in the point of time from 0.16 ± 0.03 mg/ml (n = 9) to 0.30 ± 0.04 mg/ ml by 87.5% (p = 0.016, n = 10) at the fortieth min, from 0.15 ± 0.02 mg/ml (n = 9) to 0.32 ± 0.07 mg/ml by 113.33% (p = 0.019, n = 9) at the forty-fifth min, from 0.14 ± 0.03 mg/ ml (n = 9) to 0.27 ± 0.03 mg/ml by 92.86% (p = 0.012, n = 10) at the fiftieth min, and from 0.13 ± 0.04 mg/ ml (n = 9) to 0.26 ± 0.03 mg/ml by 100.00% (p = 0.013, n = 10) at the fifty-fifth min respectively. These results suggest that RE can promote colonic mucus secretion. While pretreatment of the tissues with ketotifen 10 min prior to RE, the content of mucin reduced significantly by 31.83% (p = 0.002, n = 10) within 20 mins of drug treatment compared with treatment RE alone. All these results suggest that the RE-induced secretion of mucin-2 was partially mediated by mast cell activation.

### Identification of rhubarb and intestinal allergy

In order to authenticate the relationship between the increase of intestinal mucus secretion and defecation caused by RE and intestinal allergic reaction, IgE and IgA in colon tissue were detected by ELISA and DAB staining. As the Supplementary Figure 2 shown, pretreatment with RE at doses of 3 g/kg, 6 g/kg and 9 g/kg with gavage for 3 days, the concentration of the IgE in the colon tissue was an increasing trend by 31.7%, 49.35% and 46.05% (F = 13.78, p < 0.0001). To further confirm that RE induce IgE releasing from mast cell, treatment with ketotifen at the dose of 1 mg/kg alone, the concentration of the IgE in the colon tissue was slightly reduced compared with control group. After treatment with ketotifen 1 h later and addition with RE at the dose of 6 g/kg, it was found that the content of IgE in colon tissues is increased significantly about 30.85%. Interestingly, the ratio of CD79a positive cell for B-cell markers and its DAB mean intensity have the same trend as IgE change as shown in Supplementary Fig. 3.

### Effect of RE on serum cytokines in rats

**Figure 5:**
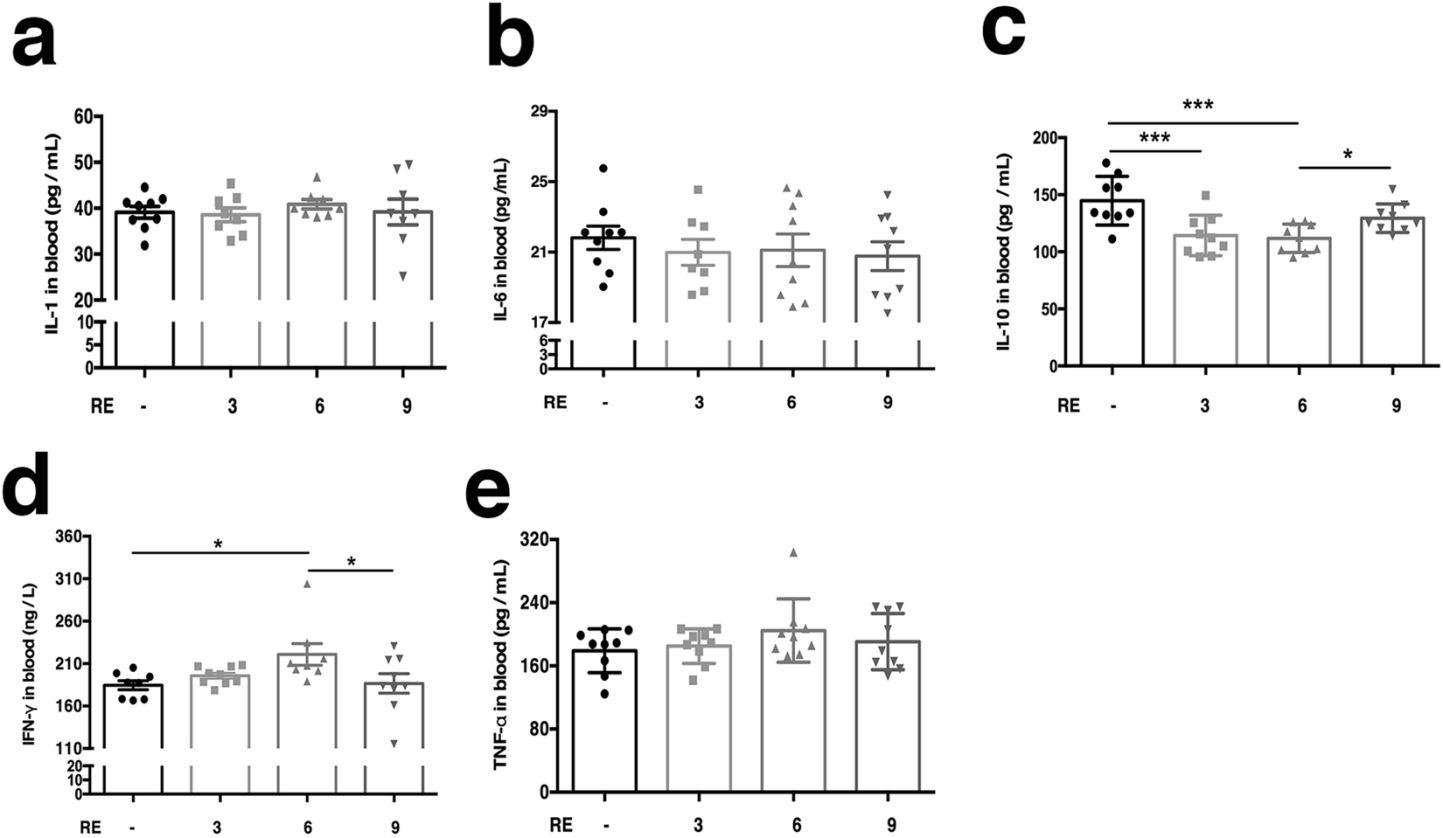
Effects of RE on changes of serum cytokine. (**a**–**e**) Concentration-response relationship for RE-evoked changes of IL-1 (F = 0.348, P > 0.05), IL-6 (F = 0.326, P > 0.05), IL-10 (F = 7.756, P = 0.0005), TNF-α (F = 1.048, P > 0.05) and INF-ɣ (F = 3.236, P = 0.036). Data are presented as the mean ± S.E.M. *P < 0.05, **P < 0.01.

Numerous studies have shown that mucus secretion is mediated by serum cytokines^19,20^. To test to what extent the cytokines involve in the process of the intragastric administration at doses of 3 g/kg, 6 g/kg, and 9 g/kg for three days. As shown in Fig. 5, there is no significant change in the content of IL-1and IL-6 in serum. The content of TNF-α exhibited an increasing trend, but with no obviously difference compared with the control group (p > 0.05). There was a significant increase of the content of IFN-γ from 184.5 ± 5.35 ng/L(n = 8) in the control group to 220.9 ± 12.76 ng/l by 19.72% (p = 0.010, n = 8) 193.6 ± 4.11 ng/L in the group with 6 g/kg RE treated, but with no obviously change by5.72% (p > 0.05, n = 8) and 1.13% (p > 0.05, n = 9) in the groups treated with 3 g/kg and 9 g/kg RE treated, respectively. At same time, the content of IL-10 was visibly reduced from 144.8 ± 7.09 pg/mL (n = 8) in the control group to 114.3 ± 5.91 pg/mL about 21.06% (p = 0.002, n = 9), 111.8 ± 4.16 pg/ml about of 22.79% (p = 0.004, n = 9) at RE doses of 3 g/kg and 6 g/kg, respectively. In contrast, the content of IL-10 was not down-regulated in dose of with RE at 9 g/kg (Fig. 5c). This result suggested RE could induce / restrain the changes of some cytokines such as IFN-γ and IL-10, which may involve in the process of regulating mucus secretion.

## Discussion

Rhubarb has long been used to treat constipation, and the earliest record can be found in *Shen Nong’s Herbal Classic*, which is currently still used in the preparation of herbal laxatives for thousands of years. However, it remains unknown how a laxative effect of rhubarb act on the colon tissue to regulate mucin secretion. This present study aimed to investigate the mechanisms underlying RE-induced mucus secretion *in vivo* and *ex vivo*.

It has been reported that the secretion of mucus is accompanied with water^5,21^. In clinical practice, the water content in stool will increase after applying rhubarb for 6–12 hours, though such short term rhubarb application (once/day for 3 days) will not cause dehydration. The side effects of rhubarb is limited due to the application of rhubarb in clinic is usually less than a week; and long-term use anthraquinone laxatives may lead to melanosis coli, as was shown in many studies^22–24^. Anthraquinones, as the main active ingredients of the Chinese rhubarb, tomato leaves and radix polygoni multiflori, are usually considered to be the chemical basis of the purgative activity of rhubarb. The associated regulatory mechanism might involve two aspects, one of which is the stimulation and irritation of the gastrointestinal tract, which promotes colonic peristalsis and accelerates colonic transit significantly^25^; on the other hand, it can increase paracellular permeability and result in an enhancement of the water content in the colon lumen^26,27^. Our previous study found that emodin induces chloride secretion in rat distal colon through activation of mast cells and enteric neurons^7^. However, the mechnism of rhubarb-induced mucus secretion remain obscure. Our study reveals that treatment with RE results in the aggregation and activation of mast cells, increase histamine release and chymase, markers of mast cells. Degranulation of mast cells causes the release of mast cell mediators, which stimulate mucus secretion by directly acting on goblet cells^28^. The specialized function of GCs is the synthesis and secretion of mucus. Mucin-2 is a component of the intestinal mucus, especially in the colon. As mucus play a vital role in the protection of the underlying epithelium, any quantitative change in mucus secretion may modify this defensive barrier. Mucus is a viscous gel composed mainly of water (90% or more), with small amounts of salts, carbohydrates, lipids and mucins^29^. It was recorded in the Chinese Compendium of Medica that RE has the function of moistening the intestines and promoting bowel movements. Depending on the goal of the research, the identification of GCs may be relevant. Particle tracking after the oral administration of RE in rats, for instance, showed clearly that the number of GCs increased, and the content of mucus were also increased. These findings suggested that mast cells participate in increasing the colonic water content to soothe constipation or cause diarrhoea with the protective mechanisms^30^. It is deduced that RE induced-mucus secretion, which could be partially blocked by ketotifen, a mast cell membrane stabilizer. IgE is the lowest level of total antibodies in normal environment^31^. The level of IgE was significantly increased in serum in the case of food allergy and intestinal parasite infection, which was closely related to the occurrence of allergic symptoms^32^. IgA plays a key role in the immune function of intestinal mucosa. Intestinal tract stimulated by various antigens and cytokines produced by mucosal T cells, accelerated the conversion of B cells to IgA cells^33^. Simultaneously, IgA is involved in the regulation of allergic reaction^34^. It can increase the output of IgA and has a strong neutralizing effect to inhibit allergic reaction^35^. Local IgE and IgA levels increased as Supplementary Figs. 2, 3. Degranulation of mucosal mast cells in the gut triggers a type I hypersensitivity response, which is characterized by powerful propulsive muscular contractions and hypersecretion that underlie a diarrhea state^36^.

**Figure 6:**
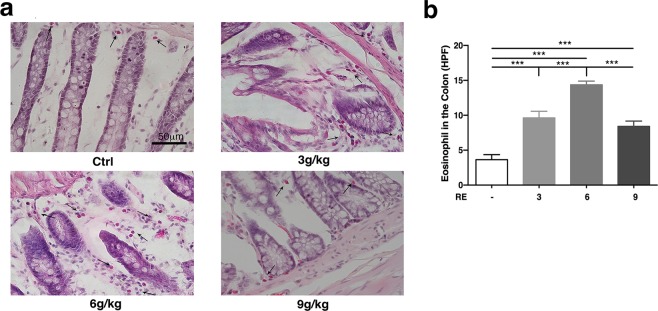
Effect of RE on Eosinophils in the colon. (**a**) RE-evoked eosinophil accumulation by H&E staining of the rat colon. (**b**) The bar graph illustrates the changes in eosinophils number with different doses of RE alone. (F = 33.61, P < 0.0001) Data are presented as the mean ± S.E.M. ***P < 0.001. Scale as 50 µm.

There is mounting evidence for interactions between the mucus secretion and enteric neurons, and immune cells^37^. Mast cells are closely associated with nerve fibers in the intestine, because of the proximity of mast cells and neural fibers, spontaneous release of mast cell mediators modulates neural reflex programs, which regulate goblet cell function^38–40^. As an important role in anaphylaxis, mast cell could quickly degranulate and release some bioactive components including chemokines, which cause smooth muscle contraction, increase in permeability and eosinophils aggregation in the lamina propria mucosa^41^. ACh and histamine are important neurotransmitters in the submucosal plexus that mediate intestinal secretion^42,43^. As seen in Fig. 1e, Fig. 3c and Fig. 6, in addition to mast cells, eosinophils also accumulated with the administration of RE, which might limit the activity of basophils in many ways and relieve allergic reactions^44^. We consider that Ach and histamine from mast cells may precipitate mucus secretion via secretagogues mucus, which might contribute to the laxative effect, which can be partly inhibited by ketotifen.

**Figure 7:**
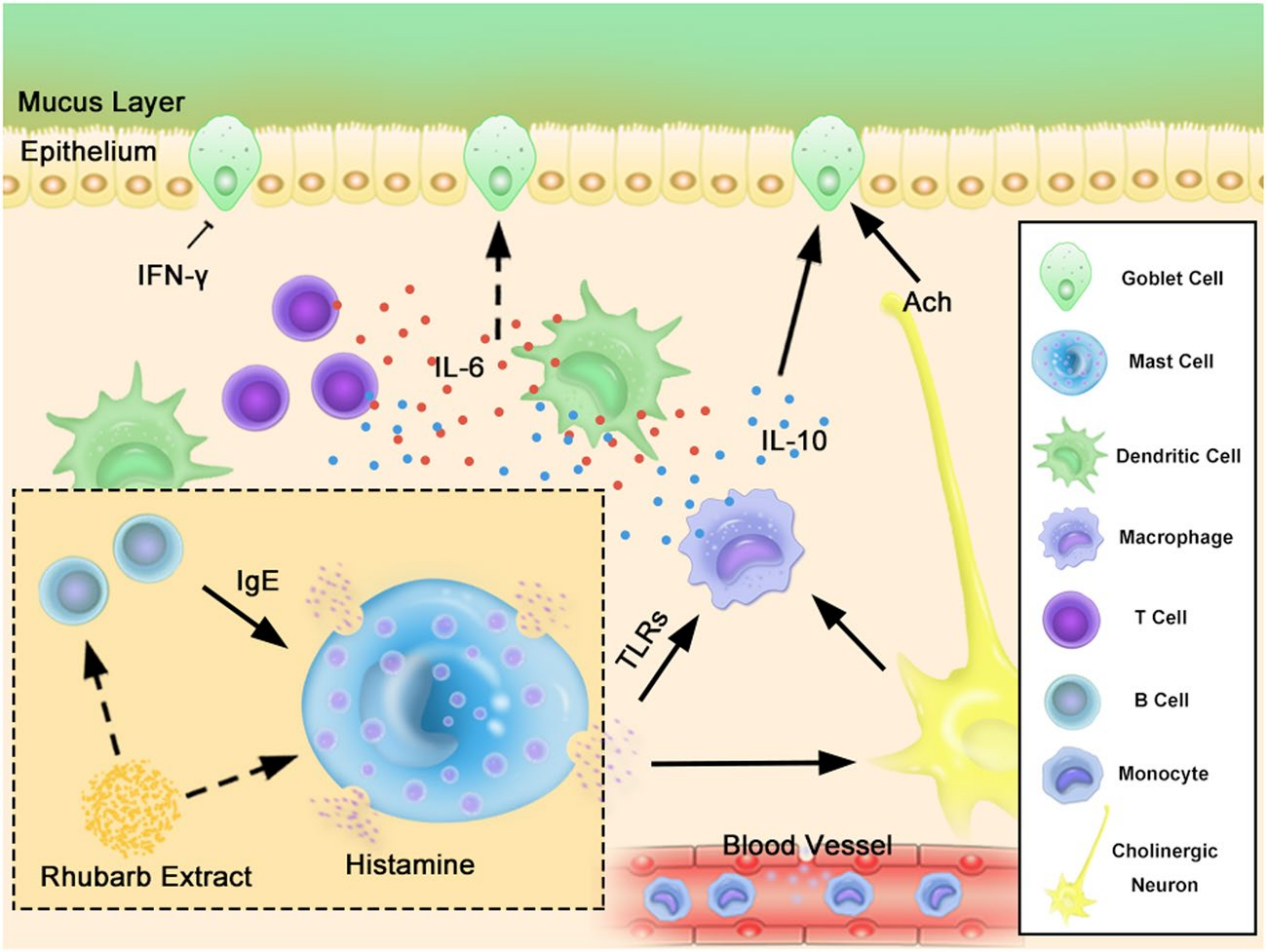
An illustration of the working hypothesis of Rhubarb-Evoke Mucus Secretion in colon. RE may stimulates mast cells to release histamine, which binds to the HR on cholinergic neurons and leads to ACh release. ACh and histamine might induce the release of IL-10 and IL-6. IL-10 could increase mucin secretion through the IL-10 receptor on GCs. IL-6 may also lead to an enhancement of mucin secretion, which may be caused by down-regulation via IFN-γ. In the figure, solid arrows represent direct excitation, dotted arrows represent indirect excitation, the T indicates inhibition.

Some interleukins are pro-inflammatory factors, such as TNF-α, IL-1 and IL-6, which play a role in the acute response. IL-6 is an important modulator in maintaining the balance between Th1 and Th2 cell effector functions. IL-10 directly relieves endoplasmic reticulum stress, promotes GC differentiation, and increases the biosynthesis and secretion of mucin-2, depending on activation of STAT1 and STAT3 during inflammation^45^. Previous reports have shown that IL-10 plays a vital role in the stimulation and maintenance of tolerance to benign allergens. In allergic rhinitis and asthma, airway IL-10 expression is reduced. In turn, this reduction leads to inflammation in response to non-harmful allergens^46^. In our experiments, there was a significant reduction in the level of IL-10 in the groups treated with 3 g/kg and 6 g/kg RE, which suggests that rhubarb caused a slight allergic reaction. However, IL-1, IL-6 and TNF-α exhibited no differences compared to the control group. Interestingly, IFN-γ increased in the 6 g/kg group. It was also reported that IL-6 signalling via membrane-bound IL-6R leads to activation of the immune system mainly through anti-inflammatory pathways in response to invading pathogens, in addition to the pro-inflammatory mechanism. The data presented herein suggest that different doses of RE caused different types of hypersensitivity or that IL-6 also participated in two mechanisms caused by the action of RE. Moreover, IL-6 has an direct/indirect regulatory effect on mucus secretion^47^ (Fig. 7).

Summarizing, the question of the *in vivo* and *ex vivo* significance of the direct effect of RE on mucus secretion must be raised. It can be postulated that changes of cytokines and mucus hypersecretion depend on RE-induced mast cell degranulation of colon and submucosal cholinergic neurons and partly non-neuronal pathway.

## Materials and Methods

### Animals and experimental design

All animal protocols followed the guidelines established by the National Institutes of Health and were approved by the Animal Care and Use Committee of Capital Medical University (IRB number: AEEI-2016-079). Male Sprague-Dawley (SD) rats (Laboratory Animal Services Center, Capital Medical University), weight ranging from 220 to 250 g (6 weeks old), had free access to standard rodent laboratory food and water until the day of the experiments. A total of 110 rats were randomly divided into different groups just as the RE therapeutic groups (3 g/kg, 6 g/kg, and 9 g/kg body weight), the control group treated with physiological saline and ketotifen treatment group. All drugs were given via intragastric administration for three days in the *in vivo* experiments. One group of rats was fed ketotifen fumarate tablets at an oral dose of 1 mg/kg 1 h before the administration of RE, and one group of rats was fed the ketotifen fumarate tablets alone. In this study, the animal were measured daily for its body weight, food intake and defecation volume. The animals were killed by cervical dislocation. Forty rats were used in the incubation test and the colon perfusion test *in vitr*o

### Drugs

Polygonum *multiflorum* rhubarb roots were purchased from Beijing Tong Ren Tang, Beijing, China. As described previously^7^, the air-dried roots were powdered, extracted by soaking for 2 h and boiling gently for 2 h and stored at 4 °C until use, and the samples were authenticated by Prof. Wen Wang, a botanist at Xuanwu Hospital in Beijing, China. The extract was diluted to 1 g/ml RE. Ketotifen fumarate was produced by Sigma (USA, Lot number: 080M1565V) and Tokyo Chemical Industry (TCI, Lot number: K0048). Krebs-Henseit solution (K-HS) includes the following ingredients (in mM) at pH 7.4: NaCl, 117; KCl, 4.5, CaCl_2_, 2.5; MgCl_2_, 1.2; NaHCO_3_, 24.8; KH_2_PO_4_, 1.2; and glucose, 11.1. (Supplementary Table 1)

### Faecal pellet output and water content

The rats were kept individually in stainless steel metabolism crates in an environmentally controlled (27 ± 2 °C) room, the bottoms of which remained open and in a moist environment to minimize the risk of water evaporation and coprophagia. Faecal samples were collected after 12 h, weighed (wet weight), desiccated under natural ventilation (37 °C, 24 h), and weighed again (dry weight). The faecal water content was calculated according to the formula:

$${\rm{Water}}\,{\rm{content}}( \% )=\frac{{\rm{wet}}\,{\rm{weight}}-{\rm{dry}}\,{\rm{weight}}}{{\rm{wet}}\,{\rm{weight}}}\times 100 \% $$

### Histopathology and immunohistochemistry

The isolated distal colon (DC, 1.5 cm to anus) samples were cut along the mesenteric border and flushed with Krebs–Henseleit solution (K–HS). Tissue specimens were fixed overnight with 4% paraformaldehyde (PFA), embedded in optimal cutting temperature (OCT) or paraffin, sliced at a 5 μm step thickness (Leica, CM1850, St. Gallen, Switzerland) and air-dried for 24 h at room temperature. The sections were immersed in a beaker containing citrate buffer (0.01 M, pH 6.0) and boiled at 95–100 °C in a microwave for 15 min for antigen retrieval. Slices were stained with 3,3′-Diaminobenzidine (DAB), Periodic Acid-Schiff/Alcian blue (PAS/AB) and haematoxylin after incubation with an anti-mast cell CD117 antibody, according to the product descriptions (Zhongshan, PV-6001, China, and Genmed Scientifics, Inc., GMS30062.4, USA). For immunofluorescence, the following steps were performed. After being washed with phosphate-buffered saline (PBS) for 5 min, the sections were incubated with 5% normal donkey serum in 0.3% Triton X-100 in PBS for 30 min at room temperature and then incubated with primary antibodies overnight at 4 °C. Subsequently, the sections were washed with PBS with Tween 20 (PBST) and incubated with the appropriate secondary antibodies at room temperature for 2 h. 4,6-Diamidino-2-phenylindole (DAPI) was used to stain the cell nuclei. Finally, the specimens were mounted with anti-fade glycerol buffer. Images were captured by using a laser confocal TCSSP5 microscope. The antibodies against histamine receptors were purchased from Abgent (H_1_ receptor: AF3587a, H_2_ receptor: AF2455a), the antibody against CD117 was purchased from Thermo Fisher Scientific (MA-170079), and the antibody against mucin-2 was purchased from Santa Cruz (Sc-15334). (Supplementary Table 1)

### Western blotting

Colon samples were homogenized in cold lysis buffer with protease inhibitor cocktail (Roche, Switzerland). The extracts were subjected to electrophoresis in polyacrylamide gels, transferred to nitrocellulose (NC) membranes (Millipore, USA) and blocked with non-fat milk dissolved in Tris-buffered saline (TBS) for 1 h. The NC membranes were incubated with primary antibodies to detect endogenous levels of total β-tubulin protein (Gxybio, P1014) and mast cell chymase (Abcam, Ab2377) overnight at 4 °C and then for 2 h at room temperature, followed by incubation with the appropriate secondary antibodies for 2 h at room temperature. The blots were visualized by using an Odyssey Infrared Imager (LI-COR, NE, USA). (Supplementary Table 1)

### Quantification of colonic mucus levels

The amount of mucus secreted from the colonic segments *in vitro* was evaluated as previously standard procedures detailed in described^48^. Segments of the colon (approximately 1 cm in length) were equilibrated in Ussing chambers, with 950 mL/L O_2_ and 50 mL/L CO_2_ in K-HS at 37 °C for 15 min. The tissues were then incubated with Kreb’s solution with an qual volume of saline was as the control, RE at the different dosage of 20 μg/mL, 40 μg/mL and 80 μg/mL L, ketotifen at the concentration of 100 μM or pretreatment with ketotifen 10 min then add RE (40 μg/mL) for 40 min. Then, the tissues samples were collected for measurements.

### Enzyme-linked immunosorbent assay

The contents of histamine, ACh and mucin-2 released from the colonic segments *in vitro* were measured by means of ELISA as previously described^49^. Faeces were soaked overnight in PBS at 4 °C and centrifuged at 8000 rpm. The supernatants were constant volume and samples of the same volume were collected to measure the concentration of histamine in the faeces. Serum was obtained by centrifugation after blood collection and was used to measure the concentrations of IFN-γ, TNF-α, IL-1, IL-6, and IL-10. ELISA kits (Biotechnology Co., Ltd., Beijing, China) were used to measure the cytokines mentioned above according to the manufacturer’s protocols. (Supplementary Table 1)

### Colon perfusion test and protein content detection

Male SD rats weighing 250–300 g (Laboratory Animal Services Center, Capital Medical University) were housed in group cages under conditions of controlled temperature (22–24 °C) and illumination (12-h light cycle starting at 6:00 AM) before experiments are maintained on laboratory chow and water. Protocols describing the use of rats were approved by the Animal Care and Use Committee, Capital Medical University and in accordance with the National Institute of Health Guide for the Care and Use of Laboratory Animals. The rats were anaesthetized with 10% chloral hydrate at a dose of 4 mL/kg. The abdominal cavities were cut and opened along the midline incision to obtain the colons (7 cm, DC, 1.5 cm to anus). Then, the colons tissue were washed with Kreb’s solution, placed in a perfusion slot (Patent number: ZL 2015 2 0899585.5), which contained K-HS with 950 mL/L O_2_ and 50 mL/L CO_2_, and maintained at 37 °C. The intestinal segments were tied at one end and mounted with a plastic pipe made with a pipette tip at the other end. The colons segments were incubated in thermostatic baths for 15 min; then the flow rate of the peristaltic pump was adjusted to 1 mL/5 min, and the outflow solution was collected at the outlet per 5 min. The samples of the control, treatment with RE at concentration of 40 μg/mL and treatment with ketotifen (100 μM) add RE (40 μg/mL) were collected in the order listed. Ketotifen was administered after three rounds of outflow solution were collected, when the colons have adapted to the environment. The outflow solution was collected into Eppendorf tubes and analysed with a Bradford protein concentration kit (Beyotime, P0006, China), as described in the manufacturer’s protocol. (Supplementary Table 1)

### Data presentation and analysis

Slices were stained with haematoxylin and eosin (H&E). The granules in eosinophils were dyed red, and positive cells were observed tissue sections from at least three tissues of five animals. All tissue specimens stained for HE, PAS/AB or CD117 were taken at a 200× magnification, sampling a total area of 2.5 mm^2^ of each slide at least four but generally six random images for microscopic evaluation of the intestinal structures and cells by an experienced pathologist in a blinded manner. The colour intensity of the NIS-Elements BR 4.10 software was set to find the target in the slices and analyse the number and chromaticity of stained cells. GCs were also counted using this method after staining with PAS/AB.

Images displayed CD79a positive cells were analyzed using the dedicated software, TissueQuest (TissueGnostics GmbH, Vienna, Austria), which can provide the algorithms for DAB positive cell authentication. The software is able to set cutoffs automatically using a class 2 cutoff algorithm according to the standard of non-reactive control primary antibodies. Or cutoffs can be identified by the operator, that is to say, background and signal intensity could be analyzed automatically using the software. Tissue virtual microscopy analyses were performed using the soft system that combines detailed morphologic information offered by microscopy with the scientific accuracy of multichannel flow cytometry. Results of the imaging analyses were expressed as total number of CD79a positive cells, and total number of effective cells. Then, it was possible to accurately quantify the percentage of cells positive for CD79a markers in each analyzed tissue samples in a fast and no bias scanning. Cell density was calculated as total number of the cells divided by total sample area in square millimeters. The same cutoff values were maintained for each marker and comparison to minimize error rate. 0.5 mm × 0.5 mm of six casual fields were analyzed for each sample. There are at least four animals in each group.

All values were expressed as the means ± standard errors of the mean (S.E.M.), and *n* is the number of animals in each experiment. All data were analyzed using the GraphPad Prism 5.0 software package (GraphPad Software Inc., San Diego, CA, USA). Differences among groups were analyzed by one-way analysis of variance (ANOVA) with Dunnett’s post-hoc test. A *P* value less than 0.05 was considered statistically significant.

## Supplementary information

Supplementary Fig1

Supplementary Fig2

Supplementary Fig3(2)

Table S1

Dataset Figure S3b

## Data Availability

The data in this study generated during and/or analyzed during the current study and supplementary information are available from the corresponding author on reasonable request.
