# Supplementary material for: Rhubarb-Evoke Mucus Secretion through Aggregation and Degranulation of Mast Cell in the Colon of Rat: In vivo and ex vivo studies
Source: Sci Rep. 2019 Dec 18;9:19375. doi: 10.1038/s41598-019-55937-7 (PMC6920142; doi:10.1038/s41598-019-55937-7)
Supplement: Supplementary file 1 — Supplementary Fig1 [file 41598_2019_55937_MOESM1_ESM.pdf]

Rhubarb-Evoke Mucus Secretion through Aggregation and Degranulation of Mast Cell in the Colon of Rat: In vivo and ex vivo studies

Di Wu[1, 2], Xiaowei Xue[3], Chenchen Gao[1], Yuehong Liu[4], Tiantian Wang[1], Lisheng Li[5], Xuehong Tong[5], Feng Li[6], Jingdong Xu[1*]

[1]Department of Physiology and Pathophysiology, School of Basic Medical Science, Capital Medical University, Beijing, 100069, China;

[2] Key laboratory of Carcinogenesis and Translational Research (Ministry of Education/Beijing), Department of Interventional Therapy, Peking University Cancer Hospital & Institute, Beijing, 100142, China;

[3] Department of Pathology, Peking Union Medical College Hospital, Chinese Academy of Medical Sciences & Peking Union Medical College Beijing, 100730, China;

[4] Department of Radiology, Xuanwu Hospital, Capital Medical University, Beijing, 100053, China;

[5]Experimental Center for Basic Medical Teaching, School of Basic Medical Science, Capital Medical University, Beijing, 100069, China;

[6] Department of Neurobiology, School of Basic Medical Science, Capital Medical University, Beijing 100069, China.

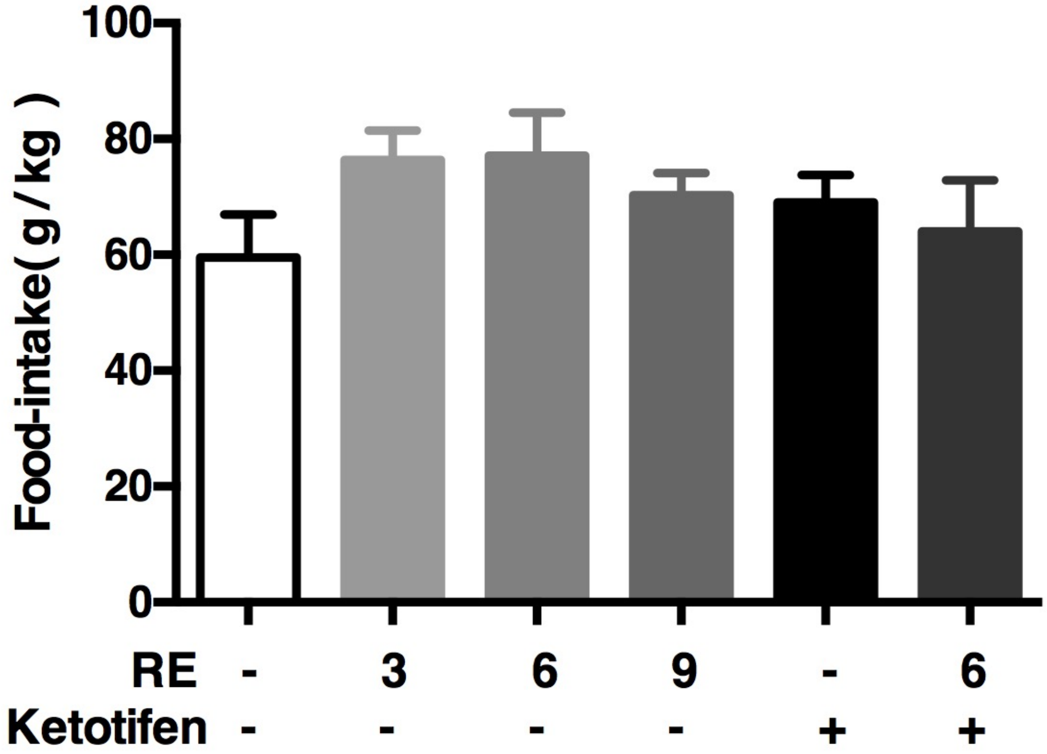

Supplementary Figure 1. The bar graph illustrates daily food-intake according to rat body weight in different groups. All data has slight changes, but with no statistical significance. Data are presented as the mean ± S.E.M. (F=1.161, P=0.338) .
