# Supplementary material for: Rhubarb-Evoke Mucus Secretion through Aggregation and Degranulation of Mast Cell in the Colon of Rat: In vivo and ex vivo studies
Source: Sci Rep. 2019 Dec 18;9:19375. doi: 10.1038/s41598-019-55937-7 (PMC6920142; doi:10.1038/s41598-019-55937-7)
Supplement: Supplementary file 3 — Supplementary Fig3(2) [file 41598_2019_55937_MOESM3_ESM.pdf]

Rhubarb-Evoke Mucus Secretion through Aggregation and Degranulation of Mast Cell in the Colon of Rat: In vivo and ex vivo studies

Di Wu[1, 2], Xiaowei Xue[3], Chenchen Gao[1], Yuehong Liu[4], Tiantian Wang[1], Lisheng Li[5], Xuehong Tong[5], Feng Li[6], Jingdong Xu[1*]

[1]Department of Physiology and Pathophysiology, School of Basic Medical Science, Capital Medical University, Beijing, 100069, China;

[2] Key laboratory of Carcinogenesis and Translational Research (Ministry of Education/Beijing), Department of Interventional Therapy, Peking University Cancer Hospital & Institute, Beijing, 100142, China;

[3] Department of Pathology, Peking Union Medical College Hospital, Chinese Academy of Medical Sciences & Peking Union Medical College Beijing, 100730, China;

[4] Department of Radiology, Xuanwu Hospital, Capital Medical University, Beijing, 100053, China;

[5]Experimental Center for Basic Medical Teaching, School of Basic Medical Science, Capital Medical University, Beijing, 100069, China;

[6] Department of Neurobiology, School of Basic Medical Science, Capital Medical University, Beijing 100069, China.

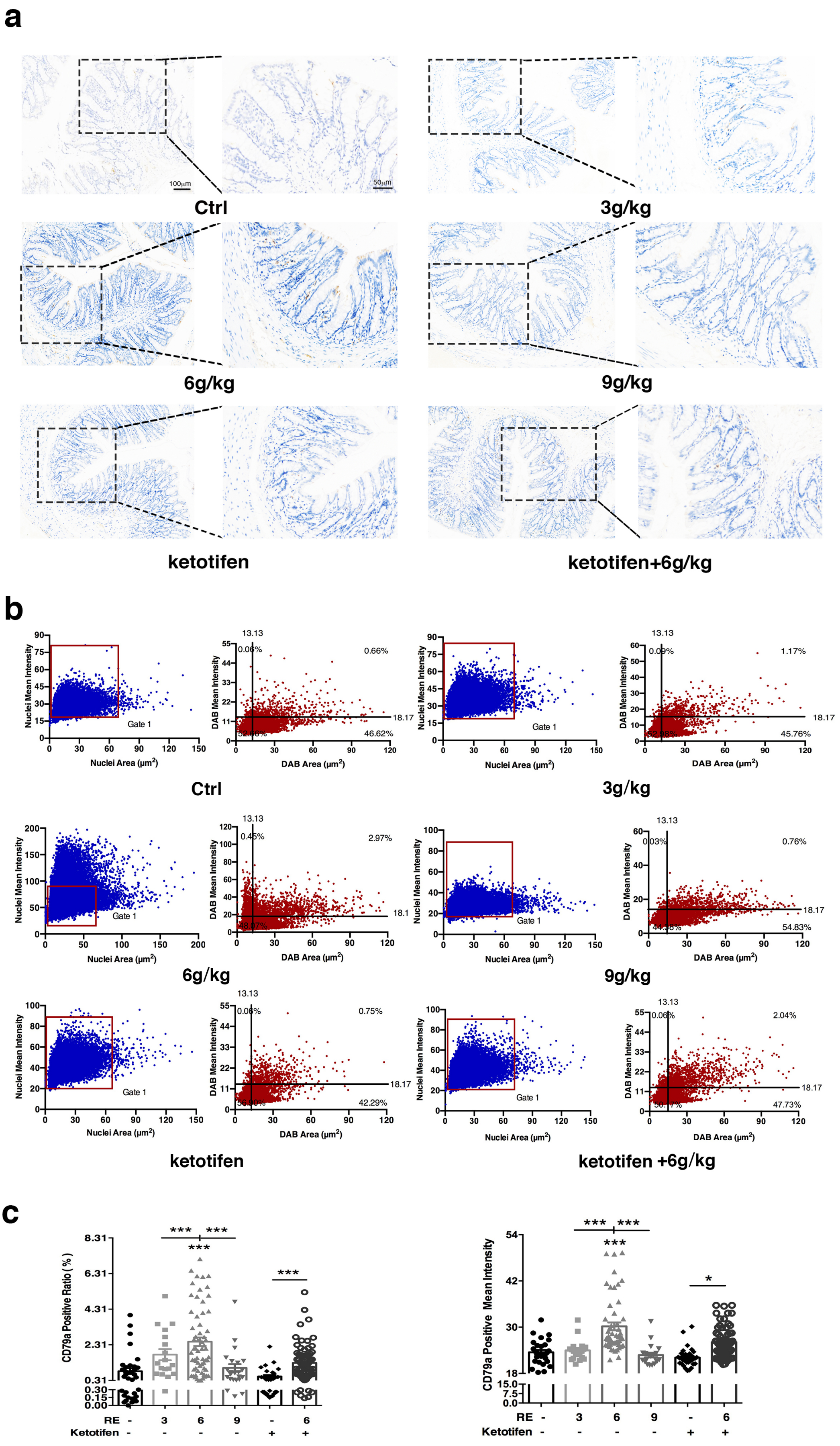
a
100μm
50μm
Ctrl
3g/kg
6g/kg
9g/kg
ketotifen
ketotifen+6g/kg
b
Nuclei Mean Intensity
Nuclei Area (μm^2)
Gate 1
DAB Mean Intensity
DAB Area (μm^2)
13.13
18.17
Ctrl
3g/kg
6g/kg
9g/kg
ketotifen
ketotifen +6g/kg
c
CD79a Positive Ratio (%)
CD79a Positive Mean Intensity
RE - 3 6 9 - 6
Ketotifen - - - - + +
***
*

Supplementary Figure 3. Immunohistochemical localization and quantification of CD79a expression. Automatically using TissueQuest software. a. CD79a positivity with DAB staining. Stainings were analyzed using a TissueFAXS microscopic system (version 3.5.5, TissueGnostics, Vienna, Austria). All colonic tissues came from the same part of the colon in rats. b. Every stainings of figure a was scanned and automatically analyzed using TissueQuest software (version 4.0.1.0127, TissueGnostics). The blue scatter plot as the mean intensity of nuclei unit area. Gate 1 as the effective cells calculated automatically by software. The red scatter plot as CD79a positive and the mean intensity of CD79a positive cells area. c. The bar graph illustrates the CD79a positive ratio ($F=14.11$, $P<0.0001$) and CD79a mean intensity in the different grougs ($F=17.41$, $P<0.0001$) . Data represents mean ± S.E.M., *$P< 0.05$ ***$P< 0.001$. Bar as 100μm, 50μm.
