# Supplementary material for: Rhubarb-Evoke Mucus Secretion through Aggregation and Degranulation of Mast Cell in the Colon of Rat: In vivo and ex vivo studies
Source: Sci Rep. 2019 Dec 18;9:19375. doi: 10.1038/s41598-019-55937-7 (PMC6920142; doi:10.1038/s41598-019-55937-7)
Supplement: Supplementary file 4 — Table S1 [file 41598_2019_55937_MOESM4_ESM.pdf]

Rhubarb-Evoke Mucus Secretion through Aggregation and Degranulation of Mast Cell in the Colon of Rat: In vivo and ex vivo studies

Di Wu[1, 2], Xiaowei Xue[3], Chenchen Gao[1], Yuehong Liu[4], Tiantian Wang[1], Lisheng Li[5], Xuehong Tong[5], Feng Li[6], Jingdong Xu[1*]

[1]Department of Physiology and Pathophysiology, School of Basic Medical Science, Capital Medical University, Beijing, 100069, China;

[2] Key laboratory of Carcinogenesis and Translational Research (Ministry of Education/Beijing), Department of Interventional Therapy, Peking University Cancer Hospital & Institute, Beijing, 100142, China;

[3] Department of Pathology, Peking Union Medical College Hospital, Chinese Academy of Medical Sciences & Peking Union Medical College Beijing, 100730, China;

[4] Department of Radiology, Xuanwu Hospital, Capital Medical University, Beijing, 100053, China;

[5]Experimental Center for Basic Medical Teaching, School of Basic Medical Science, Capital Medical University, Beijing, 100069, China;

[6] Department of Neurobiology, School of Basic Medical Science, Capital Medical University, Beijing 100069, China.

Supplementary Table 1

REAGENT	RESOURCE or SOURCE	IDENTIFIER
**Triton-X 100**	Chemical Industry	
**Bradford protein concentration determination kit**	Beyotime	P0006
**Ketotifen fumarate**	Sigma-Aldrich	K2628-100MG
**AB/PAS stain kit**	Abcam	ab245886
**Antibody**	**RESOURCE or SOURCE**	**IDENTIFIER**
**Donkey-anti- DAPI**	GENMED	
**Rabbit anti- Mucin-2**	Santa Cruz	Sc-15334
**Rabbit anti- β-tubulin**	Gxybio	P1014
**Rabbit anti- Chymotrypsin**	Abcam	Ab2377
**Goat anti- HR1**	Abgent	AF3587a
**Goat anti- HR2**	Abgent	AF2455a
**Mice anti-CD117**	Thermo Fisher	MA-170079
**Goat anti- IgA**	abcam	ab199001
**Critical Commercial Elisa kits Assays**	**RESOURCE or SOURCE**	**IDENTIFIER**
**Rat IL-1**	RGB& CHN	RGB-60018R
**Rat IL-6**	RGB& CHN	RGB-60023R
**Rat IL-10**	RGB& CHN	RGB-60026R
**Rat TNF-α**	RGB& CHN	RGB-60080R
**Rat IFN-γ**	RGB& CHN	RGB-60073R
**Rat Ach**	RGB& CHN	RGB-60083R
**Rat MUC2**	RGB& CHN	RGB-60310R
**Rat His**	RGB& CHN	RGB-60351R
**Rat 5-HT**	RGB& CHN	RGB-60087R
**Rat IgE**	RGB& CHN	RGB-60039R

**Experimental Models: Organisms/Strains**	**RESOURCE or SOURCE**	**IDENTIFIER**
**Rat：Sprague Dawley（SD）Male**	Laboratory/Capital Medical University	IRB number: AEEI-2016-079
**Software and Algorithms**	**RESOURCE or SOURCE**	**IDENTIFIER**
**Prism 5.0**	Graphpad	https://www.graphpad.com/
**StrataQuest v5.0**	TissueGnostics	http://www.tissuegnostics.com/en/products/analysing-software/strataquest
**Image-Pro 6.0**	NIH	https://imagej.nih.gov/ij

CONTACT FOR REAGENT AND RESOURCE SHARING

Further information and requests for resources and reagents should be directed to and will be fulfilled by Jingdong Xu (xujingdong@163.com).
